# A cochlear progenitor pool influences patterning of the mammalian sensory epithelium via MYBL2

**DOI:** 10.1242/dev.202635

**Published:** 2024-09-10

**Authors:** Caryl A. Young, Emily Burt, Vidhya Munnamalai

**Affiliations:** ^1^ Graduate School of Biomedical Science and Engineering, University of Maine, Orono, ME 04469, USA; ^2^ The Jackson Laboratory, Bar Harbor, ME 04609, USA; ^3^ Molecular Biosciences, The University of Kansas, Lawrence, KS 66045, USA

**Keywords:** Cochlea, Sensory epithelium, Progenitors, Boundary, Support cells

## Abstract

During embryonic development, Wnt signaling influences both proliferation and sensory formation in the cochlea. How this dual nature of Wnt signaling is coordinated is unknown. In this study, we define a novel role for a Wnt-regulated gene, *Mybl2*, which was already known to be important for proliferation, in determining the size and patterning of the sensory epithelium in the murine cochlea. Using a quantitative spatial analysis approach and analyzing *Mybl2* loss-of-function, we show that *Mybl2* promoted proliferation in the inner sulcus domain but limited the size of the sensory domain by influencing their adjoining boundary position via *Jag1* regulation during development. *Mybl2* loss-of-function simultaneously decreased proliferation in the inner sulcus and increased the size of the sensory domain, resulting in a wider sensory epithelium with ectopic inner hair cell formation during late embryonic stages. These data suggest that progenitor cells in the inner sulcus determine boundary formation and pattern the sensory epithelium via MYBL2.

## Teaser

**Summary:** *Mybl2* is a Wnt-regulated transcription factor expressed in the developing inner sulcus domain of the cochlea that influences boundary formation between progenitors and the sensory domain, thereby determining the size of the mature sensory epithelium.

## INTRODUCTION

The sensory epithelium of the cochlea, the organ of Corti, has a highly specialized organization containing one row of sound-detecting inner hair cells (IHCs) and three rows of sound-amplifying outer hair cells (OHCs) across its radial axis. Its proper organization confers auditory processing by guiding afferent and efferent innervation to target IHCs and OHCs. The sensory epithelium is flanked by the inner sulcus (IS) (also known as Kölliker's organ during development) along the medial edge of the cochlea, and outer sulcus (OS) epithelia on the lateral edge of the organ. Proper innervation of the sensory epithelium is dependent on the correct placement of epithelial boundaries. However, the molecular mechanisms that determine boundary formation are unclear. Several studies suggest that multiple signaling pathways influence the formation of the medial versus the lateral compartments and domains of the cochlea (Basch et al., 2016; Ellis et al., 2019; Hayashi et al., 2007; Huh et al., 2012; Hwang et al., 2010; Jansson et al., 2019; Munnamalai and Fekete, 2016, 2020; Munnamalai et al., 2012; Ohyama et al., 2010; Oliver et al., 2021; Petrovic et al., 2015).

Our previous studies have shown that, during development, Wnt signaling specified neural cell fates in the chicken cochlea, or influenced the formation of the medial compartment, which we define here as comprising the developing IS and the medial sensory (MS) domains in the mouse cochlea (Munnamalai and Fekete, 2016; Munnamalai et al., 2017; Oliver et al., 2021). We have also shown that the Wnt secretion enzyme PORCN was enriched on the medial half of the cochlea between mouse embryonic day (E)13.5 and E14.5 (Oliver et al., 2021) when the IS, MS, lateral sensory (LS) and OS domains were established; thus, WNT ligand secretion is optimally positioned to promote the formation of the medial IS and MS domains, especially because WNTs are classical short-range morphogens (Routledge and Scholpp, 2019). In the intestine, WNTs are secreted from the progenitor niche and are only dispersed via cell division (Farin et al., 2016). As in several developing systems, activation of Wnt signaling in the cochlea stimulated cell cycle re-entry (Jacques et al., 2012; Liu et al., 2022a; Samarajeewa et al., 2018, 2019). Several studies have shown that Wnt signaling also regulates *Jag1* or *Serrate1* expression in the mouse and chicken cochleas, respectively, which suggests that there is conservation in Wnt signaling (Jacques et al., 2012; Munnamalai and Fekete, 2016; Munnamalai et al., 2017). How the Wnt pathway regulates both proliferation and sensory specification simultaneously is unknown.

Our recent studies have shown that the SOX2-positive sensory domain undergoes refinement to be repositioned medially on E12.5 to the center of the cochlear duct floor by E14.5 (Thompson et al., 2023). The SOX2-positive sensory domain will continue to form the sensory epithelium. Upon differentiation, support cells retain SOX2 expression, whereas the hair cells (HCs) do not (Dabdoub et al., 2008). During early otic stages, the Wnt pathway positions the neurosensory domain in the inner ear (Żak and Daudet, 2021). Similarly, in this study, we investigate potential molecular mechanisms via an uncharacterized gene in the cochlea, *Mybl2*, by which the Wnt pathway can simultaneously regulate proliferation in the IS domain and determine the size of the JAG1-mediated, SOX2-positive sensory domain in the developing mouse cochlea by influencing their adjoining boundary on E14.5.

## RESULTS

**Fig. 1.**
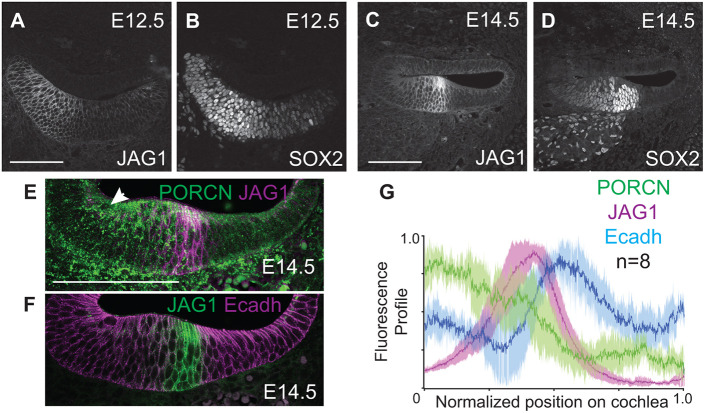
**The sensory domain is refined between E12.5 and E14.5.** (A,B) JAG1 and SOX2 were enriched at the medial edge of the cochlear duct on E12.5. (C,D) By E14.5, JAG1 and SOX2 were centrally repositioned. (E) PORCN was enriched in the medial half of the cochlea. JAG1 was expressed within the PORCN domain on E14.5. Arrow shows the absence of JAG1 in the IS domain. (F) JAG1 and E-cadherin have complementary, non-overlapping expression on E14.5. (G) Spatial profile analysis of PORCN, E-cadherin and JAG1 expression on E14.5 (*n*=8 cochleas). Scale bars: 100 μm.

To determine how the Wnt pathway influences boundary formation between the IS and the sensory domains, we compared the relative spatial position of the prosensory domain on E12.5 and E14.5 by immunolabeling for JAG1 and SOX2 (Fig. 1A-D). On E12.5, JAG1 was positioned on the medial edge of the cochlea (Fig. 1A). As JAG1-Notch signaling regulates SOX2 expression and sensory epithelial formation (Kiernan et al., 2001, 2006), the SOX2-positive sensory domain was also located medially (Fig. 1B). By E14.5, both JAG1 and SOX2 expression were refined to the center of the cochlear duct floor (Fig. 1C,D). How the medial JAG1, SOX2-positive sensory boundary is redefined during development is unknown. To determine the spatial relationship between Wnt signaling via PORCN expression and Wnt-regulated JAG1 expression on E14.5, we spatially analyzed PORCN, JAG1 and E-cadherin by co-immunolabeling E14.5 cochlear sections. PORCN was enriched in the medial half of the cochlea, and JAG1 was expressed within the PORCN domain (Fig. 1E). However, JAG1 expression was absent in the medial region where PORCN expression was present (Fig. 1E, arrow). As expected at this stage, E-cadherin was enriched in the LS domain (Fig. 1F) (Chacon-Heszele et al., 2012; Whitlon, 1993), where JAG1 was absent. Quantitative spatial profiling (Fig. 1G) revealed that, although JAG1 lies within the PORCN domain, there must be an unknown factor that suppressed JAG1 expression in the IS domain. Spatial analysis also supported that the LS domain showed neither PORCN nor JAG1 expression, where E-cadherin expression peaked (Fig. 1G).

Studies showed that *Jag1*, a direct Wnt target gene in hair follicles (Estrach et al., 2006), was positively regulated by Wnt activation in E12.5 cochleas (Jacques et al., 2012; Munnamalai and Fekete, 2016). Temporal positive Wnt regulation of *Jag1* was consistent on E12.5 when we analyzed JAG1 expression in *Emx2Cre;*β*-catenin* (β-*cat*; also known as *Ctnnb1*) conditional knockout (cKO) cochleas (Fig. S1) as *Emx2* was expressed in the cochlea by E12.5 (Holley et al., 2010) (Fig. S1A). In control cochleas, JAG1 was expressed medially, but was downregulated in *Emx2Cre;*β*-cat* cKOs (Fig. S1B,C). Total JAG1 fluorescence decreased by 71±8.8% (mean±s.e.m., *P*-value=1.04e-7) in the *Emx2Cre;*β*-cat* cKOs relative to littermate controls (Fig. S1D). However, by E14.5 JAG1 was no longer expressed at the medial edge, despite the expression of PORCN (Fig. 1E,G), which suggested that PORCN/WNT regulation of JAG1 expression was dynamic (Munnamalai and Fekete, 2013, 2016; Oliver et al., 2021). This observation was consistent with our previous studies showing that *Jag1* responsivity to Wnt activation was dependent on the developmental stage. A 6 h treatment of E12.5 cochleas *in vitro* by CHIR99021, a GSK-3β inhibitor that activates the Wnt pathway, showed an upregulation of *Jag1*. However, when explants were treated 1 day later, *Jag1* expression was suppressed (Munnamalai and Fekete, 2016).

**Fig. 2.**
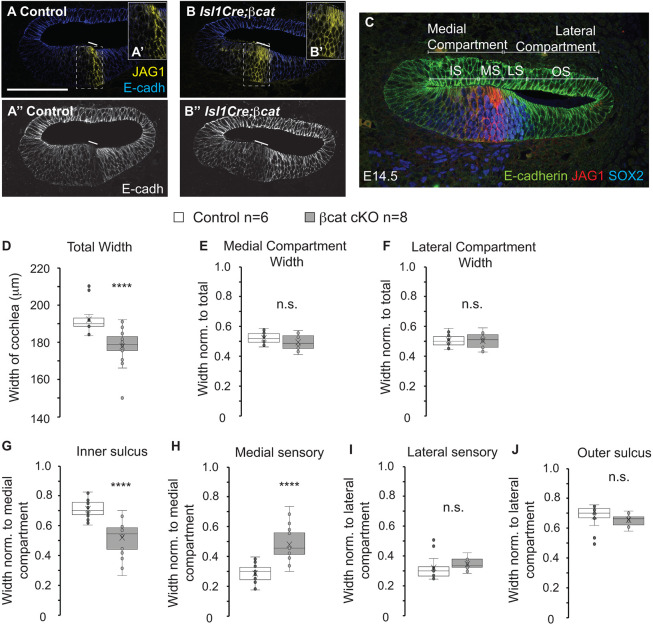
**JAG1 is spatiotemporally regulated by Wnt signaling.** (A) Immunolabeling for JAG1 and E-cadherin in E14.5 control cochlea. (A′) High magnification of MS domain (dashed boxed area in A). (A″) E-cadherin was absent in the MS domain and enriched in the LS domain. (B) JAG1 expression in *Isl1Cre*;β*-cat* cKO cochlea on E14.5 was expanded. (B′) High magnification of expanded MS domain (dashed boxed area in B). (B″) E-cadherin was downregulated in the region of JAG1 expansion. Solid lines in A,A″,B,B″ mark the MS domain. (C) Indication of domains and markers used. JAG1 labels the MS domain and defines the IS-MS boundary. E-cadherin is enriched in the LS domain. SOX2 is enriched in the sensory domain (MS+LS). The lateral boundary of SOX2 and E-cadherin segregate the LS and OS domains. (D-J) Quantification of domain sizes in control and *Isl1Cre;*β*-cat* cKOs on E14.5 (control: *N*=6, *n*=15 sections; β*-cat* cKO: *N*=8, *n*=19 sections). ****P<1e-4 (two-tailed Student's *t*-test was performed for each quantification and Bon Ferroni correction factor was applied for multiple comparisons for determining significance). n.s., not significant. (D) Total width: *P*-value=4.88e-5. (E) Medial compartment width: *P*-value=0.08. (F) Lateral compartment width: *P*-value=0.89. (G) Inner sulcus: *P*-value=2.02e-6. (H) Medial sensory: *P*-value=2.02e-6. (I) Lateral sensory: *P*-value=0.21. (J) Outer sulcus: *P*-value=0.21. Box plots show median values (middle bars), mean (X), and first to third interquartile ranges (boxes); whiskers indicate 1.5× the interquartile ranges; dots indicate data points and whiskers indicate standard deviation. Scale bar: 100 μm (for A-C).

To determine whether JAG1 was dynamically regulated by the Wnt pathway in a spatiotemporal manner, we used *Isl1Cre* to induce later β*-cat* cKO compared with *Emx2Cre. Isl1Cre*-mediated Tdt (tdTomato) expression was not present on E12.5 but was strongly expressed in the mid turn by E14.5 (Fig. S2). Next, we analyzed JAG1 expression in E14.5 *Isl1Cre*;β*-cat* cKO cochleas (Fig. 2), the stage at which the JAG1/SOX2-positive sensory domain was refined away from the medial edge (Fig. 1C,D). In the E14.5 control cochlea, the JAG1-positive MS domain was centrally positioned in the cochlear duct floor (Fig. 2A), whereas E-cadherin was enriched in the LS domain (Fig. 2A″). In E14.5 *Isl1Cre*;β*-cat* cKO cochleas (Fig. 2B), JAG1 expression was not reduced compared with E12.5 *Emx2Cre;*β*-cat* cKO cochleas (Fig. S1). We quantified the temporal impact of β*-cat* cKO between E13.5 and E14.5 using *Isl1Cre* by measuring the widths of the radial domains in the E14.5 cochlea. To determine how each domain was influenced by temporal deletion of β*-cat* on E13.5, we measured the total radial width of the cochlea, the radial widths of the medial and lateral compartments, and the radial widths of IS, MS, LS and OS domains (Fig. 2C). The MS domain was measured using JAG1 as a marker and the IS domain width was measured from the medial boundary of the MS domain to the medial luminal edge of the cochlea. The LS domain was measured using E-cadherin and SOX2, and the OS domain was measured from the lateral SOX2 boundary to the lateral luminal edge of the cochlea. The total radial width of E14.5 control cochleas was 192.2±3.12 μm, whereas the total radial width of E14.5 *Isl1Cre*;β*-cat* cKO cochleas was significantly decreased to 177.9±3.42 μm (*P*-value=4.88e-5) (Fig. 2D). The medial and lateral compartments did not significantly change in *Isl1Cre*;β*-cat* cKO cochleas relative to controls (Fig. 2E,F). The IS and MS domains were measured and normalized to the width of the medial compartment (IS+MS). The IS domain was larger and occupied 71±3% of the medial compartment, whereas the MS domain occupied 29±3% of the medial compartment in control cochleas (Fig. 2G,H). In *Isl1Cre*;β-*cat* cKO cochleas, the IS domain was decreased in size to occupy 52±4% of the medial compartment, whereas the MS domain expanded to occupy 48±4% of the medial compartment (*P*-value=2.02e-6) (Fig. 2G,H). Thus, there was a significant increase in the size of the JAG1-positive MS domain in *Isl1Cre*;β-*cat* cKO cochleas compared with controls. This was also evident from the increase in the region in which E-cadherin expression was absent in the MS domain (Fig. 2B″). The widths of the LS and OS domains were not significantly different between control and *Isl1Cre*;β*-cat* cKO cochleas (Fig. 2I,J). The increase in size of the MS domain was at the expense of the IS domain on E14.5. This led us to hypothesize that there was a Wnt-regulated gene expressed in the IS domain on E14.5 that suppressed the expression of JAG1 to drive the refinement of JAG1 from the medial edge on E12.5 to the center of the cochlear duct floor on E14.5. As cells continue to proliferate in the IS domain, we also analyzed proliferation by immunolabeling control and *Isl1Cre;*β-*cat* cKO cochleas with Ki67, a proliferation marker (Fig. S3). There was a 24±5% (*P*-value=0.009) decrease in Ki67 fluorescence in the *Isl1Cre;*β-*cat* cKOs.

To identify a potential Wnt-regulated gene, we performed RNA-sequencing on E14.0 cochleas that were treated with CHIR99021 for 6 h. We isolated genes encoding transcription factors with a log-fold change greater than 0.5 and examined their spatial expression using *in-situ* hybridization in E14.5 cochlear tissue. Consistent with PORCN expression on E14.5, *Mybl2* encoded a transcription factor that was expressed on the medial side of the cochlea, where PORCN expression was the highest. MYBL2 is known for its role in regulating the cell cycle during development and disease (Liu et al., 2022b; Musa et al., 2017; Papetti and Augenlicht, 2011; Ward et al., 2018).

**Fig. 3.**
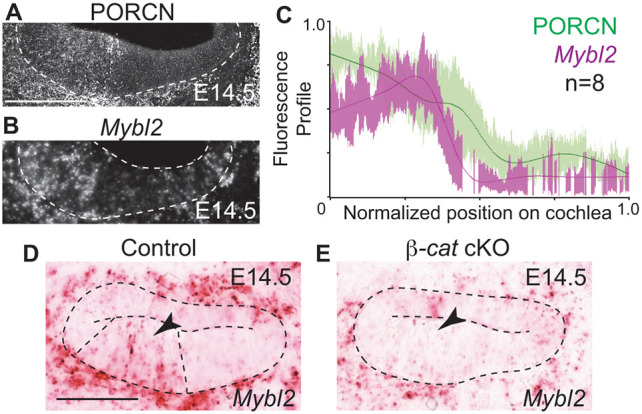
**The Wnt pathway regulates *Mybl2* in the cochlea.** (A) Immunolabeling of PORCN on E14.5 was enriched medially in wild-type cochlea. (B) *Mybl2* expression in E14.5 wild-type cochlea. (C) Quantitative spatial profile comparing PORCN and *Mybl2* expression on E14.5 (*n*=8 cochleas). (D,E) *Mybl2* expression in control E14.5 cochlea (D) and *Is1lCre;*β*-cat* cKO cochlea (E) on E14.5. Arrowhead indicates region of *Mybl2* expression. Dashed lines indicate the cochlear epithelium. Scale bars: 100 μm.

To determine whether the Wnt pathway spatially regulates *Mybl2* expression, we performed quantitative spatial analysis by immunolabeling for PORCN and *in-situ* hybridization of *Mybl2* on E14.5 wild-type cochleas (Fig. 3). PORCN was restricted to the medial half of the cochlea on E14.5 (Fig. 3A), and *Mybl2* was enriched on the medial side of the cochlear duct floor (Fig. 3B). We compared the spatial profiles of both PORCN and *Mybl2* and found that *Mybl2* lies within the PORCN expression domain (Fig. 3C), supporting that Wnt can spatially regulate *Mybl2* expression in the cochlea during development. To verify this, we examined *Mybl2* expression using *in-situ* hybridization in E14.5 *Isl1Cre*;β*-cat* cKO cochleas (Fig. 3D,E). *Mybl2* expression was present in controls (Fig. 3D, dotted lines and arrowhead), but absent in *Isl1Cre*;β*-cat* cKO cochleas (Fig. 3E, arrowhead). Therefore, Wnt signaling promoted the expression of *Mybl2* in the medial side of the cochlea.

**Fig. 4.**
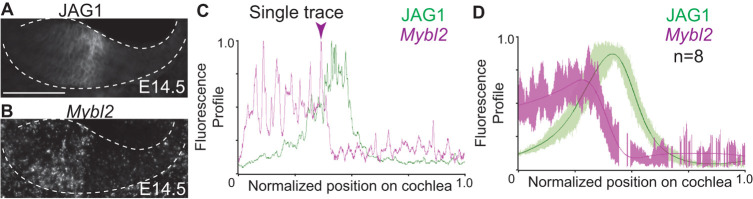
**Spatial juxtaposition of JAG1 and *Mybl2* in the E14.5 cochlea.** (A) Immunolabeling for JAG1 on E14.5. (B) *Mybl2* transcripts on E14.5 were present medially. (C) Single quantitative spatial profile plot between JAG1 and *Mybl2*. Arrowhead indicates the boundary between *Mybl2* and JAG1 enrichment. (D) Average quantitative spatial profile plot between JAG1 and *Mybl2* (*n*=8 cochleas). Scale bar: 100 μm.

The expression pattern made *Mybl2* a suitable Wnt-regulated gene encoding a transcription factor that could influence JAG1 expression on the medial side of the sensory epithelium. We performed spatial analysis of JAG1 (Fig. 4A) relative to *Mybl2* expression (Fig. 4B) in E14.5 wild-type cochlea. A single trace of JAG1 and *Mybl2* (Fig. 4A,B) showed that they were expressed in different radial domains. The lateral boundary of *Mybl2* expression abutted the medial boundary of the JAG1-positive MS domain (Fig. 4C). There was a sharp decline in *Mybl2* expression (Fig. 4C, arrowhead) where JAG1 expression peaked in the MS domain; thus, based on its expression relative to JAG1, *Mybl2* expression was in the IS domain. This spatial expression across the radial axis remained consistent in profile plots averaged across eight cochleas (Fig. 4D). Thus, *Mybl2* and JAG1 form a boundary between the IS and MS domains. We interrogated the temporally antagonistic effects of Wnt and MYBL2 on JAG1 regulation in the cochlea by comparing JAG1 fluorescence levels in the apical turns of E14.5 *Isl1Cre;*β*-cat* cKO and *Isl1Cre;Mybl2* cKO cochleas with their littermate controls (Fig. S4). In control cochleas, JAG1 had not yet undergone full refinement (Fig. S4A) and had a similar expression pattern to E12.5 (Fig. 1A). Like *Emx2Cre*;β-*cat* cKOs, we observed a 48±4.7% decrease in JAG1 fluorescent intensity in *Isl1Cre;*β-*cat* cKOs on E14.5 (Fig. S4B,C). However, when we compared *Isl1Cre;Mybl2* cKOs with littermate controls there was a 35%±5.7% increase in JAG1 fluorescence, because Wnt signaling remained active (Fig. S4D-F). This finding supports a suppressive role for MYBL2 on JAG1 expression to aid in its refinement during development.

**Fig. 5.**
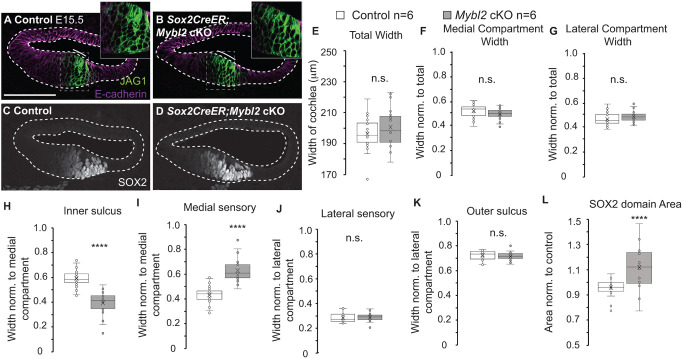
***Mybl2* specifies the size of the medial prosensory domain.** (A) Central positioning of JAG1 domain in the E15.5 control cochlea. (A′) JAG1 domain shown in high magnification for control cochleas (dashed rectangle in A). (B) E15.5 *Sox2Cre^ER^;Mybl2* cKO cochleas exhibited an expanded JAG1 domain. (B′) JAG1 domain shown in high magnification for *Sox2Cre^ER^;Mybl2* cKO cochleas (dashed rectangle in B). (C) SOX2 immunolabeling in control cochlea on E15.5. (D) SOX2 was expanded in *Sox2Cre^ER^;Mybl2* cKO cochleas. Solid lines indicate the MS domain. Dashed lines indicate the cochlear epithelium. (E-L) Quantification of domain sizes in control and *Sox2Cre^ER^;Mybl2* cKOs on E15.5 (control: *N*=6, *n*=24 sections; *Mybl2* cKO: *N*=6, *n*=24 sections). ****P<5e-4 (two-tailed Student's *t*-test was performed and Bon Ferroni correction factor was applied for multiple comparisons for determining significance). n.s., not significant. (E) Total width: *P*-value=0.17. (F) Medial compartment width: *P*-value=0.12. (G) Lateral compartment width: *P*-value=0.12. (H) Inner sulcus: *P*-value=1.28e-10. (I) Medial sensory domain: *P*-value=1.28e-10. (J) Lateral sensory domain: *P*-value=0.53. (K) Outer sulcus: *P*-value=0.53. (L) SOX2 domain area: *P*-value=1.25e-4. Box plots show median values (middle bars), mean (X), and first to third interquartile ranges (boxes); whiskers indicate 1.5× the interquartile ranges; dots indicate data points and whiskers indicate outliers. Scale bar: 100 μm.

Next, we examined the size of the JAG1-positive MS domain in *Mybl2* cKO cochleas. To test this, we generated E15.5 *Sox2Cre^ER^*;*Mybl2* cKO embryos that were induced daily with tamoxifen between E10.5 and E14.5. We used *Sox2Cre^ER^* instead of *Isl1Cre* to induce more efficient knockout of *Mybl2* along the full longitudinal axis, because *Isl1* is not expressed in the base from E12.5 (Fig. S2A) to E13.5. E15.5 cochleas were immunolabeled for JAG1 and SOX2 to measure domain sizes (Fig. 5), as previously shown in E14.5 *Isl1Cre;*β-*cat* cKO cochleas (Fig. 2). In E15.5 control cochleas, JAG1 expression was refined centrally in the cochlear duct floor (Fig. 5A). In *Sox2Cre^ER^;Mybl2* cKOs, the JAG1 domain was expanded (Fig. 5B). As SOX2 is downstream of JAG1 signaling and specifies the sensory domain, we immunolabeled for SOX2 in control and *Mybl2* cKO cochleas (Fig. 5C,D). There were no significant differences between E15.5 control and *Sox2Cre^ER^;Mybl2* cKO cochleas in the total radial width (Fig. 5E), the width of the medial compartment (Fig. 5F), or the width of the lateral compartment (Fig. 5G). When we normalized the widths of the IS and MS domains to the total width of the medial compartment, the IS domain decreased from occupying 57±3% of the medial compartment in control cochleas to 38±4% of the medial compartment in *Sox2Cre^ER^; Mybl2* cKO cochleas (Fig. 5H). The MS domain was expanded from occupying 43±3% in the control cochleas to 62±4% in *Mybl2* cKOs (*P*-value=1.28e-10) (Fig. 5I). This result followed similar trends in E14.5 *Isl1Cre;*β-*cat* cKO cochleas (Fig. 2). The widths of the LS and OS domains were not significantly affected in *Sox2Cre^ER^;Mybl2* cKO (Fig. 5J,K). We measured the area of the sensory domain by SOX2 immunolabeling and found a significant increase by 18±8% (*P*-value=1.25e-4) in the *Mybl2* cKO cochleas relative to controls (Fig. 5L). We found similar results when we used SOX2 to measure the MS domain as an alternative to JAG1 (Fig. S5). As SOX2 is also expressed in the LS, we subtracted the width of the LS using E-cadherin as a marker to obtain the width of the MS domain. In control cochleas, the SOX2-MS domain occupied 35±5% of the medial compartment, whereas the SOX2-MS domain of the *Sox2Cre^ER^;Mybl2* cKO cochleas increased to 50±5% (*P*-value=2.3e-4). Lastly, we used PRDM16 as a marker for the IS domain (Ebeid et al., 2022). The IS decreased in size from occupying 50±2% of the epithelium in control cochleas to 40±2% in the *Sox2Cre^ER^;Mybl2* cKO cochleas (*P*-value=3.26e-9) (Fig. S6). These data consistently showed an increase in the MS domain and a decrease in the IS domain in *Sox2Cre^ER^;Mybl2* cKOs. Similar results were found for domain sizes and SOX2 area using different Cre-expressing mouse lines (Fig. S7). These data suggest that, during development, MYBL2 plays a role in establishing the size of the sensory epithelium by regulating *Jag1*.

**Fig. 6.**
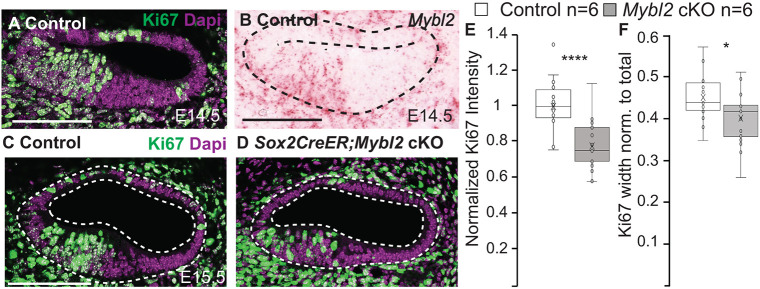
***Mybl2* regulates proliferation in the IS in the E14.5 cochlea.** (A) Ki67 immunolabels proliferating cells in the E14.5 cochlea. (B) *Mybl2* was expressed in the same region as Ki67 in A. (C) Littermate control cochlea immunolabeled for Ki67 on E15.5. (D) Ki67 labeling was decreased in *Sox2Cre^ER^;Mybl2* cKO cochleas. Dashed lines indicate the cochlear epithelium. (E) Quantification of total Ki67 fluorescence in control and *Sox2Cre^ER^;Mybl2* cKO cochleas on E15.5 (control: *N*=6, *n*=22 sections; *Mybl2* cKO: *N*=6, *n*=21 sections). *P*-value=9.33e-6 (two-tailed Student's *t*-test was performed and Bon Ferroni correction factor was applied for multiple comparisons to determine significance). (F) Quantification of Ki67-positive radial width in control and *Sox2Cre^ER^;Mybl2* cKO cochleas on E15.5 (control: *N*=6, *n*=18 sections; *Mybl2* cKO: *N*=6, *n*=19 sections). *P*-value=0.002 (two-tailed Student's *t*-test was performed and Bon Ferroni correction factor was applied for multiple comparisons to determine significance). ****P<5e-4, *P<0.01. Box plots show median values (middle bars), mean (X), and first to third interquartile ranges (boxes); whiskers indicate 1.5× the interquartile range; dots indicate data points and whiskers indicate standard deviation. Scale bars: 100 μm.

The role of MYBL2 was already known to be essential for cell proliferation (Musa et al., 2017; Papetti and Augenlicht, 2011). To rule out that the expansion of the SOX2 domain was not due to an increase in proliferation, independent of MYBL2, we investigated the impact of the loss of *Mybl2* on proliferation in the cochlea (Fig. 6). Ki67 was expressed in cells of the IS domain in control cochleas (Fig. 6A), in the same domain where *Mybl2* was expressed (Fig. 6B). On E15.5, there was a 22±6% decrease (*P*-value=9.33e-6) in Ki67 fluorescence in the IS domain of *Sox2CreER;Mybl2* cKO cochleas (Fig. 6C-E). Quantification of the Ki67-positive domain showed a decrease in the radial width of the IS domain, occupying 45±1% of the total radial width in controls compared with 40±3% in the *Sox2Cre^ER^;Mybl2* cKO cochleas (*P*-value=0.006) (Fig. 6F), which further supports that the size of the IS domain was decreased. *Isl1Cre;Mybl2* cKO also showed a similar decrease in Ki67 immunofluorescence (Fig. S8), demonstrating consistent effects using different Cre lines. Upon immunolabeling for p27^Kip1^, we found no significant difference in the width of the zone of non-proliferation between E15.5 control and *Sox2CreER;Mybl2* cKO cochleas.

**Fig. 7.**
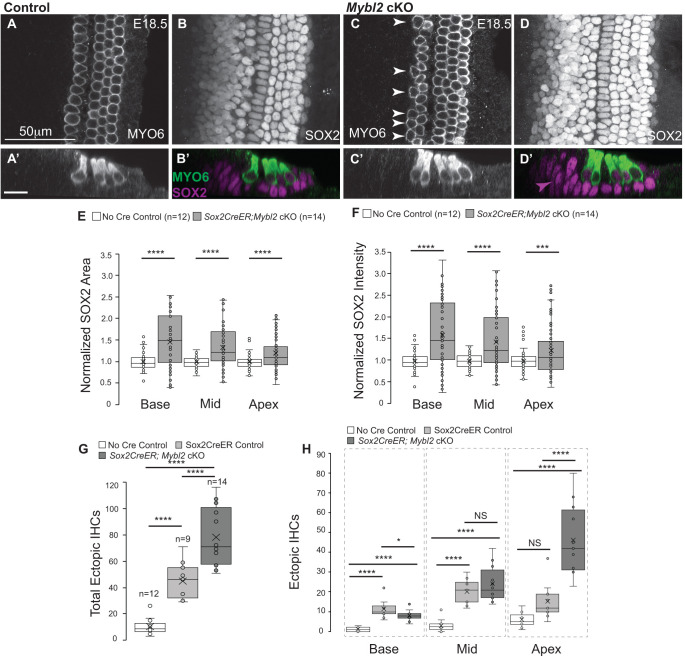
***Mybl2* is important for mature patterning of the sensory epithelium. (**A) MYO6 immunolabels the single row of IHCs and three rows of OHCs in wholemount control cochlea on E18.5. (A′) Optical cross-section of a control cochlea. (B) SOX2 immunolabels the sensory epithelium in wholemount control cochlea on E18.5. (B′) Optical cross-section of MYO6 and SOX2 labeling. (C) *Sox2Cre^ER^;Mybl2* cKOs on E18.5 show ectopic IHCs (arrowheads), labeled by MYO6. (C′) Optical cross-section of C. (D) SOX2 staining in *Sox2Cre^ER^;Mybl2* cKOs reveals an expanded sensory domain with increased SOX2 levels on E18.5. (D′) Optical cross-section of D with MYO6 (green) and SOX2 (magenta) immunolabeling. Arrowhead shows medial expansion of SOX2. (E,F) Quantification of SOX2 area and intensity in control and *Sox2Cre^ER^;Mybl2* cKO cochleas on E18.5 in base, mid and apex (control *N*=12 cochleas: *Mybl2* cKO: *N*=14 cochleas; base: *n*=69 optical sections; mid: *n*=63 optical sections; apex: *n*=72 optical sections). Two-tailed Student's *t*-test performed for statistical significance. (E) SOX2 area quantification (base: *P*-value=7.8e-9; mid: *P*-value=3.58e-7; apex: *P*-value=1.44e-4). (F) SOX2 intensity quantification (base: *P*-value=2.93e-8; mid: *P*-value=3.19e-6; apex: *P*-value=1.83e-3). (G) Quantification of total ectopic IHCs in ‘no Cre’ control cochlea, *Sox2Cre^ER^*^(+/−)^ control cochlea and *Sox2Cre^ER^;Mybl2* cKO cochleas. Tukeys test performed comparing *Sox2Cre^ER^;Mybl2* cKO (*P*-value=6e-11) and *Sox2Cre^ER^*^(+/−)^ control (*P*-value=2.04e-4) with ‘no Cre’ control cochlea, and *Sox2Cre^ER^;Mybl2* cKO (*P*-value=2.1e-4) with *Sox2Cre^ER^*^(+/−)^ control (‘no Cre’ control: *N*=12 cochleas; *Mybl2* cKO: *N*=14 cochleas; *Sox2Cre^ER^*^(+/−)^ control: *N*=9 cochleas). Bon Ferroni correction factor was applied for multiple comparisons for determining significance. (H) Quantification of IHC doublets in ‘no Cre’ control, *Sox2Cre^ER^*^(+/−)^ control and *Sox2Cre^ER^;Mybl2* cKO cochleas in the base, mid and apex. Tukeys test performed comparing *Sox2Cre^ER^;Mybl2* cKO base (*P*-value=6.9e-6) and *Sox2Cre^ER^*^(+/−)^ control base (*P*-value=2.2e-8) with ‘no Cre’ control base, and *Sox2Cre^ER^;Mybl2* cKO base (*P*-value=0.02) with *Sox2Cre^ER^*^(+/−)^ control base. Tukeys test performed comparing *Sox2Cre^ER^;Mybl2* cKO mid (*P*-value=1.8e-8) and *Sox2Cre^ER^*^(+/−)^ control mid (*P*-value=7.4e-6) with ‘no Cre’ control cochlea mid, and *Sox2Cre^ER^;Mybl2* cKO mid (*P*-value=0.39) with *Sox2Cre^ER^*^(+/−)^ control mid. Tukeys test performed comparing *Sox2Cre^ER^;Mybl2* cKO apex (*P*-value=4.8e-9) and *Sox2Cre^ER^*^(+/−)^ control apex (*P*-value=0.2) with ‘no Cre’ control cochlea apex and *Sox2Cre^ER^;Mybl2* cKO apex (*P*-value=4.5e-6) with *Sox2Cre^ER^*^(+/−)^ control apex (‘no Cre’ control: *N*=12 cochleas; *Mybl2* cKO: *N*=14 cochleas; *Sox2Cre^ER^*^(+/−)^ control: *N*=9 cochleas). Bon Ferroni correction factor was applied for multiple comparisons for determining significance. Box plots show median values (middle bars), mean (X), and first to third interquartile ranges (boxes); whiskers indicate 1.5× the interquartile range; dots indicate data points and whiskers indicate standard deviation. *P<0.05, ***P<0.001, ****P<5e-4. NS, not significant. Scale bar: 50 μm in A-D; 20 μm in A′-D′.

To determine whether the JAG1 and SOX2 boundary effects leading to an expanded sensory domain that we observed on E15.5 influenced mature cochlear patterning, we analyzed E18.5 *Sox2Cre^ER^*;*Mybl2* cKO cochleas (Fig. 7). Tamoxifen was administered daily from E10.5 until E14.5 and cochleas were harvested on E18.5. By E15.0, *Mybl2* was no longer expressed in the cochlea; therefore, tamoxifen induction was not required after E14.5. We immunolabeled wholemount cochleas for myosin VI (MYO6) (Fig. 7A,C) and SOX2 (Fig. 7B,D) to label HCs and sensory epithelium (support cells), respectively. We observed an expanded SOX2 domain and an increase in SOX2 fluorescence in E18.5 *Sox2Cre^ER^;Mybl2* cKO cochleas relative to littermate controls (Fig. 7B,D). In addition to the expanded sensory epithelium, we also observed ectopic IHCs in *Mybl2* cKO cochleas (Fig. 7C, arrowheads, and 7 C′). Optical sections from *z*-stacks on wholemount cochleas were used to quantify changes in SOX2 area and SOX2 intensity (Fig. 7A′-D′) because we observed an accumulation of SOX2-positive cells medially in the *Mybl2* cKOs, which would contribute to changes in area of the sensory epithelium (Fig. 7D′, magenta arrow). Both radial area and fluorescence of SOX2 differed in control cochleas based on longitudinal location. Thus, we quantified SOX2 in the basal, mid and apical regions (Fig. 7E,F). We found significant increases in SOX2 area and fluorescence in all regions of the cochlea, with the largest increase observed in the base and smallest increase observed in the apex of *Mybl2* cKOs compared with controls. In the base there was a 47±16% (*P*-value=7.8e-9) increase in SOX2 area, and a 59±21% (*P*-value=2.93e-8) increase in SOX2 fluorescence. In the mid-region of the cochlea, the loss of *Mybl2* resulted in a 33±12% (*P*-value=3.58e-7) increase in area, and a 43±18% (*P*-value=3.19e-6) increase in fluorescence. Lastly, in the apex, we measured a 20±11% (*P*-value=1.44e-4) increase in area and a 23±16% (*P*-value=1.8e-3) increase in fluorescence in *Mybl2* cKOs compared with control littermates. Although the average increase was smallest in the apex, we did observe maximum increases of 50% in SOX2 area and 72% in SOX2 intensity in individual sections. Thus, the loss of *Mybl2* in the apex is capable of producing larger areas of sensory epithelia. Despite previous reports that the *Sox2Cre^ER^* mice result in *Sox2* haploinsufficiency and a decrease in *Sox2* levels in cochleas relative to their ‘no Cre’ littermates (Atkinson et al., 2018); we observed a significant increase in SOX2 levels in *Sox2Cre^ER^;Mybl2* cKO cochleas, which suggests that there was a positive feed-forward loop to compensate and enhance SOX2 in *Sox2Cre^ER^;Mybl2* cKO cochleas beyond the ‘no Cre’ controls.

Although the primary effect of the loss of *Mybl2* was on the SOX2-positive sensory epithelium, we also quantified the number of IHC doublets in control and *Mybl2* cKO cochleas (Fig. 7G). We harvested E18.5 cochleas from *Sox2Cre^ER^;Mybl2* cKO embryos and their ‘no Cre’ control littermates, and also generated E18.5 cochleas from *Sox2Cre^ER^*^(+/−)^ heterozygous ‘control’ embryos. We included *Sox2Cre^ER^*^(+/−)^ cochleas as the appropriate control as previous reports showed the formation of ectopic IHCs due to *Sox2* haploinsufficiency (Atkinson et al., 2018) because excess SOX2 inhibits HC formation (Dabdoub et al., 2008). Comparing cochleas from these three groups and performing a one-way ANOVA, we found that IHC doublets increased to 78.5±6.3 in *Sox2Cre^ER^;Mybl2* cKO cochleas (*P*-value=6e-11, Tukey test) and 45±4.9 IHC doublets in *Sox2Cre^ER^*^(+/−)^ control cochleas (*P*-value=2.04e-4, Tukey test) compared with the ‘no Cre’ control cochleas that had 10.5±1.8 IHC doublets (Fig. 7G). The increase in IHC doublets was significantly higher in *Sox2Cre^ER^;Mybl2* cKO cochleas than in *Sox2Cre^ER^*^(+/−)^ control cochleas (*P*-value=2.1e-4, Tukey test). Excess SOX2 inhibits HC development, and SOX2 immunofluorescence varied longitudinally along the cochlea; thus, we binned IHC doublets in the base, mid and apex (Fig. 7H). The base ‘no Cre’ control cochleas had 1.3±0.36 IHC doublets, while the *Sox2Cre^ER^;Mybl2* cKO had 8.2±0.79. However, the *Sox2Cre^ER^*^(+/−)^ control cochleas had the largest increase to 11.5±1.4 IHC doublets in the base. The mid region of ‘no Cre’ cochleas had 3.1±0.89 IHC doublets, compared with 24.83±2.6 in the *Sox2Cre^ER^;Mybl2* cKO and 20.33±2.2 in the *Sox2Cre^ER^*^(+/−)^ cochleas. There was no significant difference in the number of mid-region IHC doublets between the *Sox2Cre^ER^;Mybl2* cKO and *Sox2Cre^ER^*^(+/−)^ cochleas. In the apex there were no significant differences in IHC doublets between the ‘no Cre’ and *Sox2Cre^ER^*^(+/−)^ control cochleas. The ‘no Cre’ control had 6.1±1.1 IHC doublets, whereas the *Sox2Cre^ER^*^(+/−)^ control had 15.4±3.2 IHC doublets. The largest increase in IHC doublets was in the apex of *Sox2Cre^ER^;Mybl2* cKO cochleas at 47.8±4.8 in the *Sox2Cre^ER^;Mybl2* cKO compared with ‘no Cre’ and *Sox2Cre^ER^*^(+/−)^ controls (*P*-value=4.8e-9, *P*-value=4.5e-6, respectively, Tukey test).

The total length of E18.5 *Mybl2* cKOs were significantly shorter by 15% compared with the ‘no Cre’ littermate controls (*P*-value=6e-7, Tukey test), and 8% shorter compared with the *Sox2Cre^ER(+/^*^−)^ control cochleas (*P*-value=0.005, Tukey test). We counted the number of aligned IHCs and the total number of IHCs (aligned+ectopic) in the ‘no Cre’ control, *Sox2Cre^ER^*^(+/−)^ control and the *Sox2Cre^ER^;Mybl2* cKO cochleas. There were no significant differences in the number of aligned IHCs/1000 μm between the three groups. If the cochlea length was decreased compared with the *Sox2Cre^ER^*^(+/−)^ control cochleas but the number of aligned IHCs did not change, we would have expected a decrease in the total number of IHCs in *Sox2Cre^ER^;Mybl2* cKO cochleas relative to *Sox2Cre^ER^* control cochleas. However, there was no significant change in the total number of IHCs/1000 μm, which suggests that the number of ectopic IHCs that were formed in the *Sox2Cre^ER^;Mybl2* cKO increased by 74% relative to the *Sox2Cre^ER^*^(+/−)^ control, despite the shortening of the cochlea by 8%. We also did not observe any change in cochlear length in E15.5 *Sox2Cre;Mybl2* cKO cochleas; thus, the increase in the MS domain size preceded any observable changes in cochlear length.

As we observed a similar expansion of JAG1 in both *Isl1Cre;*β-*cat* cKO (Fig. 2) and *Sox2Cre^ER^;Mybl2* cKOs (Fig. 5), we predicted that *Isl1Cre;*β-*cat* cKOs cochleas would also result in additional IHCs. Similar to *Mybl2* cKOs, E18.5 *Isl1Cre;*β-*cat* cKO cochleas showed additional HCs from mid to apical regions (Fig. S9). E18.5 *Isl1Cre;*β-*cat* cKO cochleas were 15% shorter compared with littermate controls (*P*-value=0.008).

## DISCUSSION

**Fig. 8.**
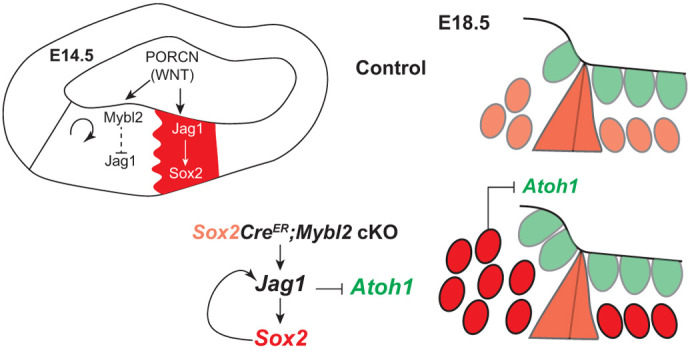
**Model for Wnt mediating roles in proliferation and patterning via regulation of *Mybl2* in the cochlea.** Wnt influences proliferation and patterning of the medial compartment via regulation of *Mybl2*. MYBL2 inhibits JAG1 expression to specify the IS and MS domains. *Mybl2* cKO results in an increase in the size of the sensory domain.

The Wnt pathway is most known for its proliferative role during development and disease (Chai et al., 2012, 2011; Jacques et al., 2012; Munnamalai and Fekete, 2013, 2016; Ng et al., 2019). In the mouse and chicken inner ears, pharmacological activation of the Wnt pathway and overexpression of WNT ligands induced proliferation (Jacques et al., 2012; Munnamalai et al., 2017; Stevens et al., 2003). Thus, the Wnt pathway holds promise for promoting regeneration in the mammalian cochlea to restore hearing (Jan et al., 2013; McLean et al., 2017; Samarajeewa et al., 2018, 2019). However, recent studies in the cochlea showed that Wnt signaling is also necessary for early sensory specification through the regulation of *Jag1*. JAG1-Notch signaling then regulates the expression of SOX2 to establish the sensory domain and, eventually, the mature sensory epithelium. Other studies have shown that Wnt signaling also promotes differentiation (Davidson et al., 2012; Shi et al., 2010, 2014) and the Wnt pathway likely has stage-dependent roles (Munnamalai and Fekete, 2013). Thus, it is important to understand the multifaceted roles underlying Wnt signaling. *Jag1* was shown to be a direct Wnt target gene (Estrach et al., 2006). Consistent with this, our previous studies and others have shown that treatment of mouse cochleas with a Wnt activator, or infection of chicken cochleas with a *Wnt-ligand*-expressing virus showed an increase in *Jag1*, or the chicken homologue, *Serrate1* (Jacques et al., 2012; Munnamalai and Fekete, 2016; Munnamalai et al., 2017). Both are genes expressed on the neural side of the cochlea (medial side in the mouse cochlea). Our previous studies have shown that, although there are several WNT ligands expressed in the cochlea, the WNT secretion enzyme (Proffitt and Virshup, 2012) PORCN is spatially enriched on the medial side, which suggests there is higher WNT secretion medially (Oliver et al., 2021). Thus, WNT secretion would be higher on the medial side to specify both proliferation and sensory specification. However, we show that on E14.5, proliferating cells in the IS are segregated from the JAG1-positive MS domain. This suggests that proliferation and sensory specification are two distinct processes that are co-regulated by separate Wnt-mediated mechanisms (Fig. 8). This is also supported by an increase in *Jag1* in response to Wnt activation after a 6 h CHIR99021 treatment on E12.5, before an increase in proliferation observed at 24 h (Munnamalai and Fekete, 2016). Given that WNTs are short-range morphogens (Farin et al., 2016; Routledge and Scholpp, 2019), spatial profiling of PORCN relative to *Mybl2*-positive proliferating cells in the IS domain and the JAG1-positive MS domain in the mouse cochlea mid-development on E14.5 show that both Ki67 and JAG1 expression lie in the PORCN expression domain; thus, are within positional range to be regulated by the Wnt pathway.

Previous studies have quantitatively shown that the SOX2 domain that was once located on the medial edge on E12.5 was gradually repositioned to the center of the cochlear duct floor by E14.5 (Thompson et al., 2023). We showed that it is during this stage that a separate pool of proliferating cells occupies the medial, IS domain where PORCN expression is enriched but is adjacent to the SOX2-positive sensory domain (Oliver et al., 2021). This led us to postulate that Wnt signals in this pool of cells, or niche, influenced the boundary between the IS domain and the sensory domain. Thus, we sought to identify the molecular mechanism by which the Wnt pathway simultaneously regulates proliferation and refines the JAG1 domain to establish the IS-sensory domain boundary. Consistent with previous studies that showed early Wnt activation led to an increase in JAG1 expression (Jacques et al., 2012; Munnamalai et al., 2017; Żak and Daudet, 2021), we showed that early deletion of β*-cat* reduced JAG1 expression (Fig. S1), whereas later deletion expanded its expression domain. These data suggest that there is an unknown Wnt-regulated gene expressed in the IS domain on E13.5-E14.5 that suppresses *Jag1* in the IS domain to refine and re-position JAG1 in the MS domain. Our Wnt-transcriptomic and *in-situ* hybridization studies revealed that *Mybl2*, a previously uncharacterized gene in the cochlea, was expressed in, and regulated proliferation in, the IS domain.

Based on quantitative spatial analysis of *Mybl2* relative to JAG1, we hypothesized that MYBL2 was expressed in the correct location to influence the formation of the medial boundary of the MS domain, while maintaining a pool of proliferating cells in the IS domain. This implies that, in addition to its role as a cell cycle regulator, MYBL2 plays a previously undiscovered function in sensory epithelial patterning by influencing the positioning of the medial sensory boundary. In support of this hypothesis, MYBL2 ENCODE ChIP-seq data showed *Jag1* to be an MYBL2 target gene (Rouillard et al., 2016). *Mybl2* cKO led to an increase in the size of the SOX2-positive sensory domain on E15.5. It is interesting to note that this increase in SOX2 area and levels occurred despite a decrease in proliferation in the IS domain of *Mybl2* cKO cochleas, suggesting that *Jag1*-mediated sensory specification is a proliferation-independent event of MYBL2. The SOX2 domain extends into the IS domain due to a relief in JAG1 suppression and a medial shift in the IS-MS domain boundary. In this way, the Wnt pathway can simultaneously regulate proliferation in the IS domain and boundary formation via *Jag1* by spatiotemporally regulating *Mybl2*, and thus determine the radial width of the sensory epithelium (Fig. 8). Interestingly, we found no significant quantitative changes in the radial widths of the lateral domains of the cochlea with the loss of *Mybl2*.

We expected that the increase in the SOX2 domain on E15.5 would leave a lasting impact on E18.5. As such, when we analyzed E18.5 *Sox2CreER;Mybl2* cKO cochleas, we found that both the size of the SOX2-positive sensory epithelium and SOX2 levels were significantly enhanced compared with ‘no Cre’ littermate control cochleas. These effects on the sensory epithelium were consistent from the basal, mid and apical regions of the cochlea. Previous studies have shown that *Sox2* haploinsufficiency was caused by the *Sox2Cre^ER^*^(+/−)^ line, resulting in a 30% decrease in *Sox2* levels compared with ‘no Cre’ littermate controls (Atkinson et al., 2018). However, in our experimental paradigm, because the conditional deletion of *Mybl2* expanded the SOX2 domain, this created a feed-forward loop to enhance *Sox2*-mediated Cre recombination, ultimately resulting in compensation and an increase of SOX2 above levels seen in the ‘no Cre’ control cochleas (Fig. 8). We observed an 8% decrease in length in the *Sox2CreER;Mybl2* cKO compared with the *Sox2Cre^ER^*^(+/−)^ control cochleas, whereas SOX2 area increased by a minimum of 20% in the apex and up to 47% in the base of the *Sox2CreER;Mybl2* cKOs. Thus, the modest changes in cochlear length would not fully account for the significantly larger changes in the E18.5 sensory epithelium. The increases in SOX2 levels of *Sox2CreER;Mybl2* cKO cochleas also indicate proliferation-independent MYBL2-mediated activity.

A shorter cochlea because of decreases in proliferation alone and no effect on signaling would only produce a shorter cochlea with a normal pattern. The changes in the IS-sensory domain boundary and sizes of domains in the absence of *Mybl2* on E15.5 when there was no change in cochlear length suggests that it is unlikely to be due to lengthening of the cochlea alone. Furthermore, during these stages PORCN expression persisted medially on E15.5 and only the patterning of the medial domains was specifically impacted in E18.5 *Mybl2* cKO cochleas.

Owing to the expanded sensory epithelium and a shift in the medial boundary of the MS domain with elevated SOX2 levels, we saw an increase in IHC doublets. However, excess SOX2 can be repressive to *Atoh1* expression (Dabdoub et al., 2008) and it is likely that the *Sox2* haploinsufficiency and the reported decreases in SOX2 levels in *Sox2Cre^ER^*^(+/−)^ cochleas was permissive to ectopic IHC formation (Atkinson et al., 2018). However, in the context of *Sox2Cre^ER^;Mybl2* cKO cochleas, we saw significant increases in SOX2 levels in the expanded sensory epithelium on E18.5. In fact, the *Sox2Cre^ER^;Mybl2* cKO cochleas would have had an even greater potential for the formation of IHCs if it were not for the possible repression of *Atoh1* by excess SOX2 (Fig. 8). This can be inferred from the inverse relationship of SOX2 levels and the number of IHC doublets along the longitudinal axis (Fig. 7F,H), which supports a repressive function of SOX2 on HC formation. This may highlight an important explanation for why it is more challenging to induce regeneration in the base relative to the apex; thus, we must be cognizant of high SOX2 levels that may be repressive when applying these findings towards regeneration. Despite the increase in SOX2 levels, we still saw a 74% greater increase of total ectopic IHCs in *Sox2Cre^ER^;Mybl2* cKO cochleas.

Similar to *Mybl2* cKOs, *Isl1Cre;*β*-cat* cKO cochleas also produced additional HCs. Our findings were similar to previous studies on *Sox2Cre^ER^;*β*-cat* cKOs, which showed that these additional HCs were of IHC fate (Jansson et al., 2019), suggesting that *Mybl2* may be the underlying downstream mechanism of the medial effects seen in β*-cat* cKOs.

In summary, the Wnt pathway is indeed capable of simultaneously regulating proliferation and sensory domain specification in a spatial manner to establish the boundary in between the MS and IS domain, via MYBL2. We also identified a previously undescribed role for MYBL2 as a transcription factor that mediates this boundary and patterning of the sensory epithelium spatially by suppressing *Jag1*. As both *Jag1* and *Mybl2* are regulated by the Wnt pathway, and one gene feeds forward to suppress another, this resembles subdomain formation by an incoherent feed-forward loop. Thus, the proliferating cells in the IS play an unexpected role in influencing patterning of the mammalian auditory sensory epithelium. One possibility for the need for such a role for the progenitors in the IS is to endow ‘non-sensory stem cell-like’ characteristics and restrict sensory specification to maintain developmental plasticity for replacing damaged or dying cells during the developmental process until the sensory epithelium has fully formed, serving as a cochlear progenitor pool, or niche with potential for acquiring sensory cell fates under the right conditions.

## MATERIALS AND METHODS

### Animal husbandry

We used β*-cat flox* [B6(Cg)-Ctnnb1^tm1Knw^/J, JAX Strain #022775, The Jackson Laboratory] and *Mybl2 flox* (B6;B6CB-Mybl2<tm2Sis>, RIKEN) mice to generate cKOs. The β-*cat* mice were crossed with *Emx2Cre* [B6.Cg-Emx2<tm2(cre)Sia>/SiaRbrc, RIKEN] mice for early cochlear cKO by E12.5, and *Isl1Cre* [Isl1^tm1(cre)Sev^/J, JAX Strain #024242, The Jackson Laboratory] for later cochlear knockout in the mid-turn by E13.5. *Emx2Cre*;β*-cat* cKO embryos were harvested on E12.5. *Isl1Cre;*β-*cat* cKO embryos were harvested on E14.5, late in the day. *Mybl2 flox* mice were crossed with *Sox2Cre^ER^* [B6;129S-^Sox2tm1(cre/ERT2)^Hoch/J JAX Strain #017593, The Jackson Laboratory] to generate cKOs. Mice were timed-mated, and the day a plug was observed was designated E0.5. Pregnant dams were administered tamoxifen (1.5 mg/10 g body weight) once daily between E10.5 and E14.5 to generate *Sox2Cre^ER^;Mybl2* cKOs. *Sox2Cre^ER^;Mybl2* cKO embryos were harvested on E15.5 and E18.5. Swiss Webster wild-type mice (Envigo, IN, USA) were used to generate E14.5 embryos for expression studies. Ai9 [B6.Cg-Gt(ROSA)26Sor^tm9(CAG-tdTomato)Hze^/J, Jax Strain #007909, The Jackson Laboratory] mice were used to show *Tdt* expression upon Cre recombination. Sequencing data at these stages did not reveal sex differences; therefore, we indiscriminately pooled data from all embryos. All experiments were performed under Institutional Animal Care and Use Committee (IACUC) protocol 18013 (PI Vidhya Munnamalai), in compliance with the US Department of Health and Human Services and reviewed by The Jackson Laboratory IACUC.

### RNA-sequencing

RNA-sequencing was performed on Swiss Webster E14.0 cochlear tissue that was treated with DMSO (vehicle) or 10 μM CHIR99021 for 6 h. Four biological replicates were prepared for control and CHIR99021-treated samples. Total RNA was phenol-chloroform extracted and purified using Qiagen mini Rnease kit. A cDNA library was prepared using the NEB Next Ultra Directional RNA library prep kit (Illumina), following manufacturer's protocols (Illumina HiSeq4000 low-input polyA-enriched RNAseq). 75 bp paired-end fragments were sequenced to 60 million read depth. Sequence reads were mapped to *Mus musculus* GRCm38.91 using the HISAT2 aligner. Library preparation and sequencing were performed at the Institute of Genome Sciences at the University of Maryland. We identified *Mybl2* from genes that encoded transcription factors with a significant log-fold change greater than 0.5. RNA-sequencing data have been deposited in GEO under accession number GSE256069.

### Histology

Embryos were decapitated and heads were fixed in 4% paraformaldehyde (PFA) (Electron Microscopy Sciences) overnight at 4°C. Embryo heads were processed through 10%, 20% and 30% sucrose solutions and placed in tissue freezing medium (General Data Healthcare) at the bottom of cryomolds and then flash frozen in liquid nitrogen. Embryos were embedded and sectioned to produce precise left-right symmetric sections to allow for precise and consistent domain quantifications. Cryofrozen tissue was sectioned at 12 µm on a Cryostar NX70 and mounted on Superfrost plus glass slides (Fisher Scientific). E18.5 wholemount cochleas were fixed in 4% PFA.

### Immunofluorescence and *in situ*-hybridization

Sectioned tissues were blocked with 2% donkey serum (Jackson ImmunoResearch) in PBS/0.5% Triton X-100 for 1 h at room temperature followed by primary antibody incubation overnight at 4°C. The following day, tissues were incubated with Alexa-conjugated secondary antibodies (1:500; A-11055, A-21082, A-21206, A-31573, A-11011 and A-21209) for 2 h at room temperature (Invitrogen) and nuclei were counterstained with DAPI (Abcam). Tissues were mounted with Fluoromount G mounting medium (Life Technologies Corporation). Primary antibodies included: rat anti-E-cadherin (1:250, ab11512, lot 1012145-3, Abcam), goat anti-Jagged1 (1:250, sc-6011, lot I2115, Santa Cruz Biotechnology), rat anti-Ki67 (1:250, 14-5698-82, lot 2196796, Invitrogen), goat-anti SOX2 (1:500, AF2018, lot KOY0421101, R&D Systems), rabbit anti-SOX2 (1:500, ab97959, lot GR3244869-1, Abcam), rabbit anti-Myosin VI (1:500, 25-6791, Proteus), rabbit anti-PORCN (1:250, PA5-43423, Invitrogen), sheep anti-PRDM16 (1:250, AF6295, R&D Systems), rabbit anti-p27^Kip1^(1:250, RB9019-P0, NeoMarkers). Antigen retrieval was performed for Ki67 immunolabeling with 10 mM sodium citrate/0.05% Tween 20 buffer at pH 6.0 for 15 min at 99°C. RNAscope for *Mybl2* was performed according to the manufacturer's protocol (Advanced Cell Diagnostics). The probe used to detect transcripts was Mm_Mybl2 (563211).

### Data acquisition and image processing

Image acquisition was performed using a Zeiss LSM800 confocal microscope at The Jackson Laboratory Microscopy core and on a brightfield/epifluorescence Olympus BX51 microscope with a Spot insight CMOS camera used to acquire images at 20× and 40×. *Z*-stacks were analyzed using FIJI software (National Institutes of Health). Measurements were made on raw image data. For figure preparation, TIFF images were processed in Adobe Photoshop and assembled in Adobe Illustrator.

### Data analysis and quantification

Spatial profile analyses were performed along the floor of the cochlear duct using the FIJI software. Profile expression data were plotted along the total widths of the respective cochleas, averaged across samples and plotted in R. The total width of the cochlea was measured from the medial edge to the lateral edge on the luminal side. Fluorescence intensity data was normalized between 0 and 1 with intensity minima at 0 and intensity maxima at 1. Profile plots were compiled across eight cochleas.

Domain size measurements were performed using FIJI. Thresholding was used to determine comparable fluorescence levels demarcating domain boundaries and the widths of the domains were measured between these boundaries. The widths of the IS and MS domains using JAG1 and SOX2 as MS markers were normalized to the width of the medial compartment of the cochlea and the widths of the OS and LS domains were normalized to the width of the lateral compartment of the cochlea. PRDM16 and Ki67 domain widths were normalized to the total width of the cochlear epithelium. JAG1 immunolabeled the MS domain, E-cadherin and SOX2 immunolabeling were used to determine the lateral boundary of the LS. The size of the total SOX2 domain and total Ki67 fluorescent levels on E15.5 was determined in FIJI by setting a minimum threshold and measuring the area and intensity of the signal, respectively. On E18.5, orthogonal projections were generated in FIJI, a threshold was set, and the total SOX2 area and intensity were measured. All area and total intensity data were normalized to the respective control mean values. The ectopic IHCs are HCs that were observed medially to the main aligned single row of IHCs and were counted along the total length of the cochlea. Wholemount images were shown as both maximum projections and orthogonal optical sections.

Quantifications were performed on a minimum of 15 sections from at least six cochleas per condition (exact number of sections and cochleas are stated in the legends of each figure). All data fit normal distributions. Statistical analysis between control and cKO cochleas was performed in R by independent two-tailed Student's *t-*test. To compare the three groups on E18.5 [‘no Cre’ control, *Sox2Cre^ER^*^(+/−)^ and *Sox2Cre^ER^;Mybl2* cKOs] we performed a one-way ANOVA, followed by a post-hoc, Tukey test to compare each group with one another. Corrected *P*-values were calculated and applied for multiple comparisons.

## Supplementary Material

10.1242/develop.202635_sup1 Supplementary information
