# Supplementary material for: A cochlear progenitor pool influences patterning of the mammalian sensory epithelium via MYBL2
Source: Development. 2024 Sep 10;151(17):dev202635. doi: 10.1242/dev.202635 (PMC11423912; doi:10.1242/dev.202635)
Supplement: Supplementary information [file develop-151-202635-s1.pdf]

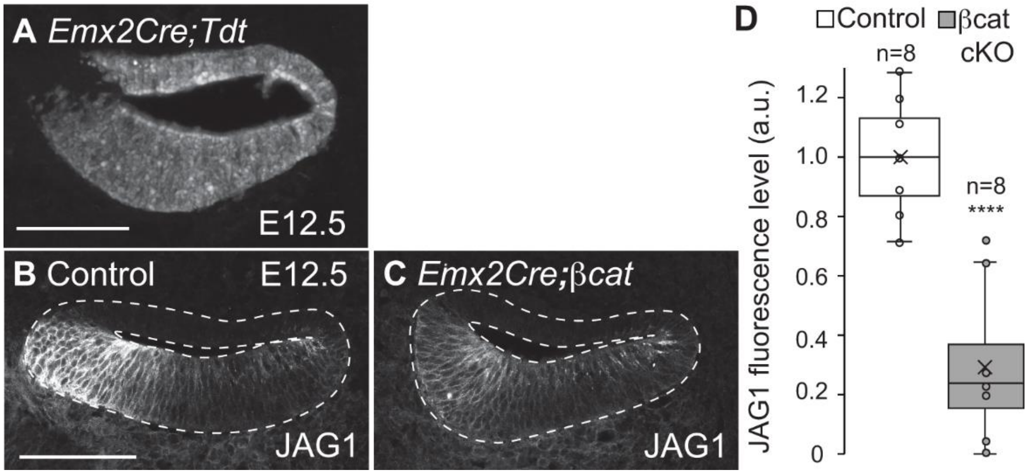

Fig. S1. JAG1 is regulated by the Wnt signaling pathway.

(A) TDT expression upon *Emx2Cre* mediated recombination on E12.5. (B, C) Control cochlea shows JAG1 expression on the medial edge, while early loss of *β-cat* resulted in a decrease in JAG1 expression on E12.5. (D) Quantification of total JAG1 intensity in control and *Emx2Cre*;*β -cat* cKOs on E12.5. (N = 8 cochleas, n = 12 sections per condition, Students two-tailed t-test p-value = 1.04e-7). Scale bar = 100µm

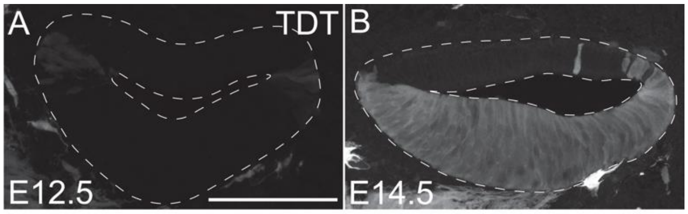

Fig. S2. *Isl1Cre* mediated recombination on E12.5 and E14.5.

(A) TDT expression is absent in the cochlea on E12.5 in *Is1lCre*$^{(+/-)}$*;Tdt* cochleas. (B) TDT expression shows Cre recombination in the mid turn of the E14.5 *Is1lCre*$^{(+/-)}$*;Tdt* cochlea. Scale bar = 100µm

Development • Supplementary information

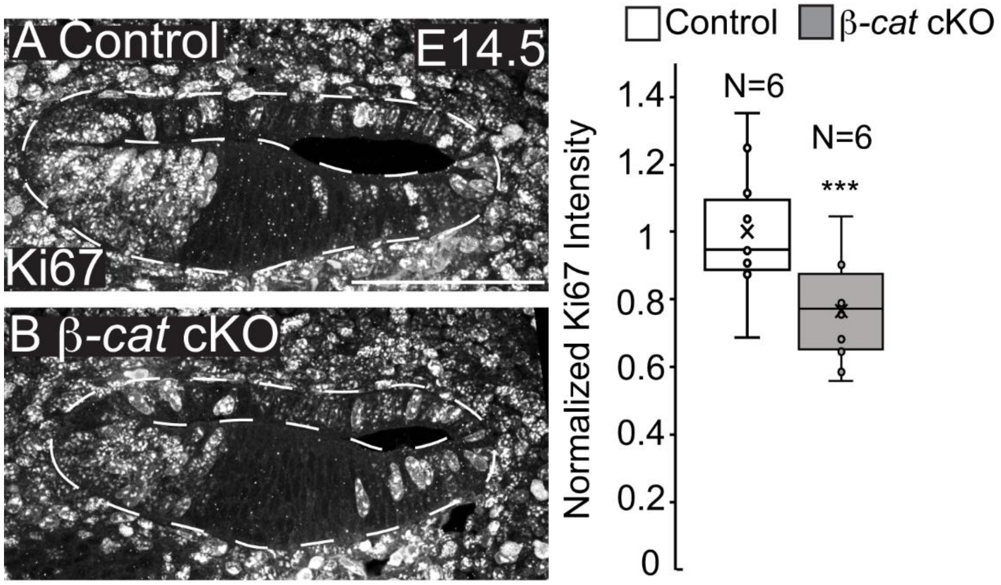

Fig. S3. Loss of *β-catenin* decreased proliferation in the IS domain.

(A) Ki67 labels proliferating cells in the cochlear epithelium in control cochlea on E14.5. (B) Ki67 is decreased in *Isl1Cre;β-cat* cKO cochlea on E14.5. (C) Quantification of total Ki67 levels in control and *Isl1Cre;β-cat* cKO cochleas on E14.5. (N =6 cochleas, 10 sections per condition, Students two-tailed t-test p-value = 0.008). Scale bar = 100μm

Development • Supplementary information

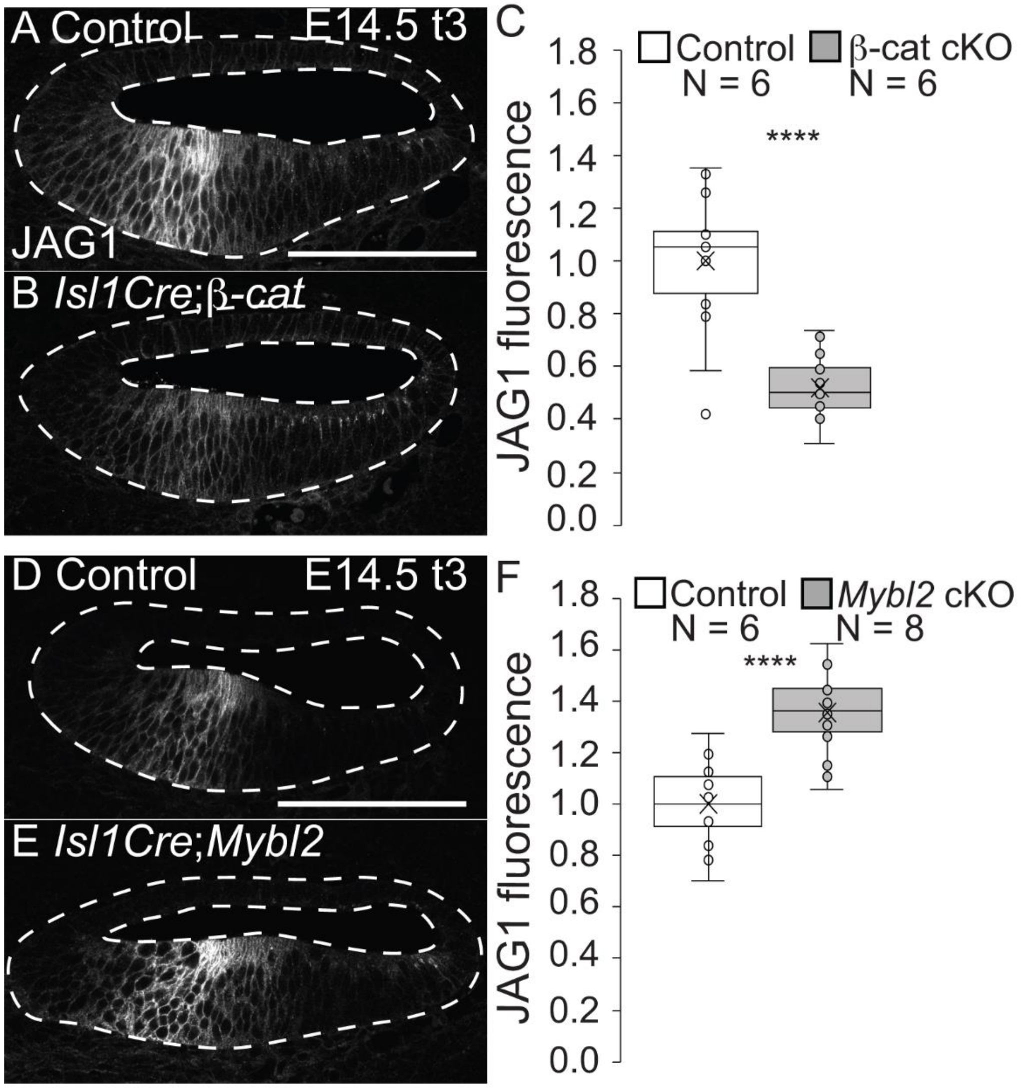

Fig. S4. JAG1 is positively regulated by Wnt signaling and suppressed by MYBL2.
(A) JAG1 expression in the apical turn of control cochlea on E14.5. (B) JAG1 levels are decreased in *Isl1Cre;β-cat* cKOs. (C) Quantification of JAG1 fluorescent intensity in apical control and *Isl1Cre;β-cat* cKOs on E14.5. (Control N = 6 cochleas, 14 sections; *β-cat* cKO N =6 cochleas, n = 16 sections, Students two-tailed t-test p-value = 4.5e-7). Scale bar = 100µm). (D) JAG1 expression in apical turn in littermate control on E14.5. (E) JAG1 levels are increased in *Isl1Cre;Mybl2* cKOs. (F) Quantification of JAG1 fluorescent intensity in apical control and *Isl1Cre;Mybl2* cKOs on E14.5. (Control N = 6 cochleas, 16 sections; *Mybl2* cKO N =8 cochleas, n = 15 sections, Students two-tailed t-test p-value = 1.7e-6). Scale bar = 100µm).

Development • Supplementary information

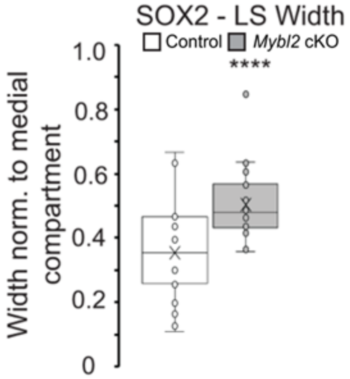

Fig. S5. SOX2 domain width is increased in Mybl2 cKOs.

Quantification of SOX2- LS width in control and *Sox2Cre*^{ER}*;Mybl2* cKO cochleas on E15.5. p- value = 2.3e-4. **** P-value<5e-4.

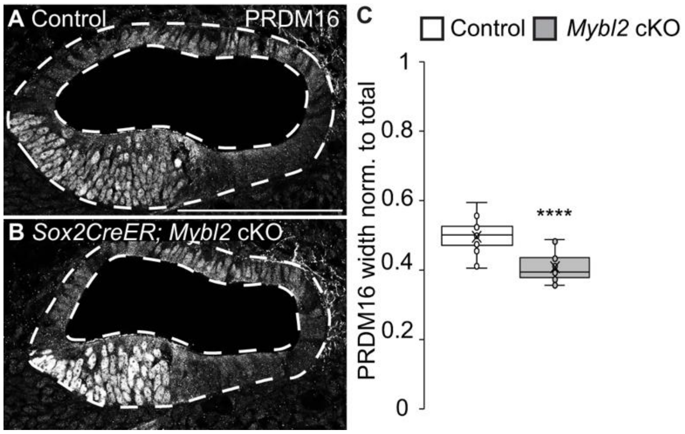

Fig. S6. The size of the PRDM16-positive domain is decreased in *Sox2Cre*^{ER}*;Mybl2* cKO cochleas on E15.5.

(A) PRDM16 is expressed in the IS domain in control cochlea on E15.5. (B) The IS domain is decreased in the *Sox2Cre*^{ER}*;Mybl2* cKOs. (C) Quantification of PRDM16 domain in control and *Sox2Cre*^{ER}*;Mybl2* cKOs. (Control N = 6, n = 24 sections; *Mybl2* cKO N = 6, n = 25 sections). Two-tailed students' t-test and Bon Ferroni correction factor were applied for multiple comparisons to determine significance. p-value = 3.26e-9. P-value < 5e-4 indicated by ****. Scale bar = 100µm

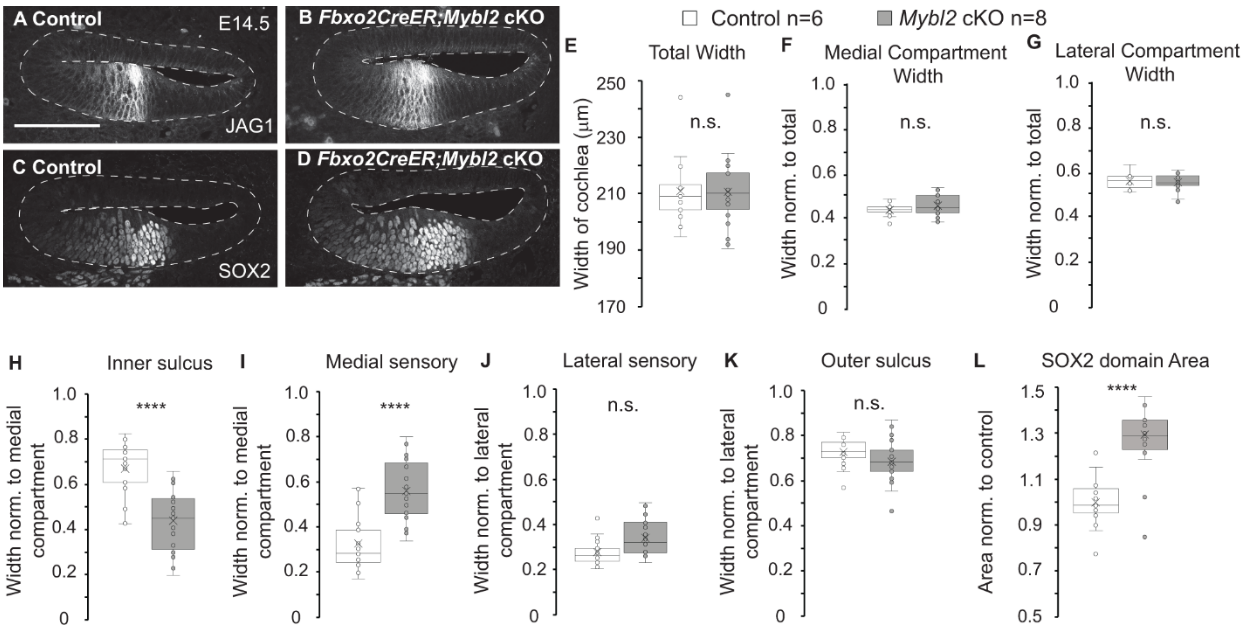

Fig. S7. *Fbxo2Cre^{ER};Mybl2* cKOs also show an increase in the MS domain width.

E14.5. (D) SOX2 labeling was expanded in *Fbxo2Cre^{ER}; Mybl2* cKO cochleas. (E- L) Quantification of domain sizes in control and *Fbxo2Cre^{ER}; Mybl2* cKOs on E14.5 (Control N =

6, n = 17 sections, *Mybl2* cKO N = 8, n = 21 sections). ****P<5e-4 (two-tailed Students t-test was performed for each quantification and Bon Ferroni correction factor was applied for multiple comparisons for determining significance. (E) Total Width was not significant, p-value = 0.98 (F-G) Medial Compartment Width, p-value = 0.14 and lateral Compartment Width, p-value =

0.60 were not significant. (H) Inner Sulcus p-value = 3.6e-6, (I) Medial Sensory domain p-value = 3.6e-6. (J) Lateral Sensory domain p-value = 0.007. (K) Outer Sulcus p-value = 0.15, (L) SOX2 domain p-value = 8.6e-6. Scale bar = 100µm

Development • Supplementary information

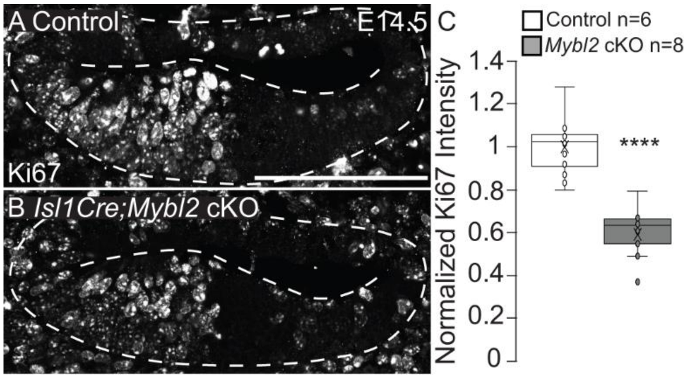

Fig. S8. *Isl1Cre;Mybl2* cKO decreased proliferation in the IS domain.

(A) Proliferating cells labeled with Ki67 in control cochlea on E14.5. (B) Ki67 labeling is decrease in *Isl1Cre;Mybl2* cKOs on E14.5. (C) Quantification of total Ki67 levels in control and *Isl1Cre;Mybl2* cKOs on E14.5. (Control N = 6 cochleas, 16 sections; *Mybl2* cKO N =8 cochleas, n = 17 sections, Students two-tailed t-test p-value = 2.5e-11). Scale bar = 100μm).

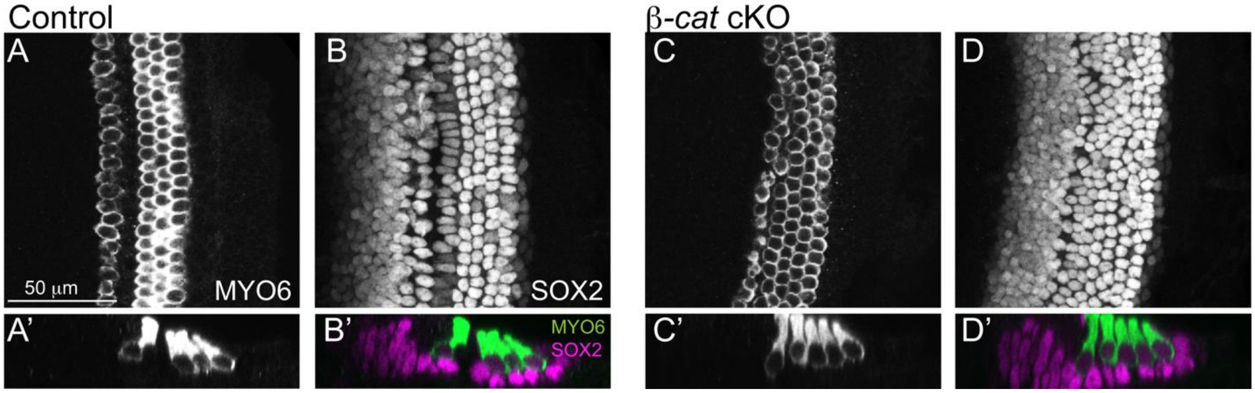

Fig. S9. Loss of β-cat between E12.5 and E14.5 impacted on radial patterning by E18.5.

(A) MYO6 immunolabels a single row of IHCs and three rows of OHCs in control cochlea on E18.5. (B) SOX2 immunolabels the supporting cells in the control cochlea. (A'-B') Optical cross-section of MYO6 and SOX2 labeling in A'-B'. (C-D) MYO6 and SOX2 immunolabeling in *Isl1Cre;β-cat* cKOs on E18.5 show additional HCs, labeled by MYO6. (C'-D') Optical cross-section of C-D.

Development • Supplementary information
